# Shared vision between fathers and daughters in family businesses: the determining factor that transforms daughters into successors

**DOI:** 10.3389/fpsyg.2015.00625

**Published:** 2015-05-29

**Authors:** Kathy K. Overbeke, Diana Bilimoria, Toni Somers

**Affiliations:** ^1^Department of Organizational Behavior, Weatherhead School of Management, Case Western Reserve UniversityCleveland, OH, USA; ^2^Deparment of Management and Information Systems, School of Business Administration, Wayne State UniversityDetroit, MI, USA

**Keywords:** vision, family business, succession, gender, daughters, women, career choice

## Abstract

Family businesses are critical to the United States economy, employing 63% of the workforce and generating 57% of GDP (University of Vermont, 2014). Family business continuity, however, remains elusive as approximately 70% of family businesses do not survive the second generation (Poza, 2013). In order to augment our understanding of how next generation leaders are chosen in family businesses, we examine daughter succession. Using a sample of pairs of family business fathers and daughters and drawing on an earlier study of the dearth of successor daughters in family businesses (Overbeke et al., 2013), we reveal that shared vision between fathers and daughters is central to daughter succession. Self-efficacy and gender norms influence shared vision and when fathers and daughters share a vision for the future of the company, daughters are likely to be transformed into successors.

## Introduction

The ability to transfer tacit and explicit knowledge throughout a successor's childhood has been recognized as a competitive advantage to family businesses (Cabrera-Suarez et al., 2001; Poza et al., 2004). Statistics revealing the dearth of daughter successors, however, suggest that daughters are either unexposed to this knowledge or their knowledge is underutilized. In 1994, only two percent of CEOs in family businesses were female including women who replaced their husbands due to death or illness and women who started their own businesses. While the number of daughters that head family businesses increased in the last decade, reaching 9.5% in 2005 (Vera and Dean, 2005), it remains surprisingly low.

The limited research on daughter succession examines the experience of daughters in family business leadership positions providing evidence of the value daughters bring to the firm (Dumas, 1989, 1990, 1992; Curimbaba, 2002; Vera and Dean, 2005; Lozano et al., 2011), gender barriers that prevent daughters from advancing their efforts in the firm (Barnes, 1988; Dumas, 1989, 1990, 1992; Hollander and Bukowitz, 1990; Iannarelli, 1992; Curimbaba, 2002; Haberman and Danes, 2007; Barrett and Moores, 2009; Jimenez, 2009; Lozano et al., 2011; Overbeke et al., 2013), and the unique benefits daughters derive from working in the firm (Cole, 1997; Vera and Dean, 2005; Haberman and Danes, 2007; Jimenez, 2009; Lozano et al., 2011). These studies amplify our understanding of successor daughters, but they also raise questions about why so few daughters reach leadership levels and the factors that enable the exceptions to become successors. This is important because the dearth of daughter successors suggests a lack of diversity in the highest levels of family business hierarchies where diversity can be a source of competitive advantage (Bilimoria et al., 2014). The historically poor rate of family business sustainability argues for an investigation into how next generation leaders are chosen and why so few daughters become successors. A meager 30% of family businesses survive from the first generation to the second (Poza, 2013). The other 70% either fail or are sold (Stalk and Foley, 2012). More daughter successors may enhance the odds of family business continuity.

The purpose of the present study is to examine drivers and barriers to daughter succession. We extend the results of our previous qualitative study of the dearth of daughter successors in family businesses (Overbeke et al., 2013) which found that gender norms, or prevailing expectations of men and women in society, blind daughters to possibilities of succession, resulting in a small supply of interested and qualified daughters who can successfully assume leadership roles. This earlier study proposed that daughters who become successors are differentiated by their confidence in their business skills, perceptions of support from influential family members, and personal visions (Boyatzis and Akrivou, 2006) that embrace family business leadership. Building on these findings, we test an empirical model based on gender norms, self-efficacy, and vision that predicts daughter succession in family owned businesses. Using a field survey of pairs of family business fathers and daughters, we examined the mediating role of vision between beliefs about daughters' efficacy as leaders of family businesses, daughters' and fathers' gender role orientations, daughters' and fathers' perceptions of sexism in society, and the outcome variable, daughter succession or intention to be a successor.

## Theory and hypotheses

**Figure 1:**
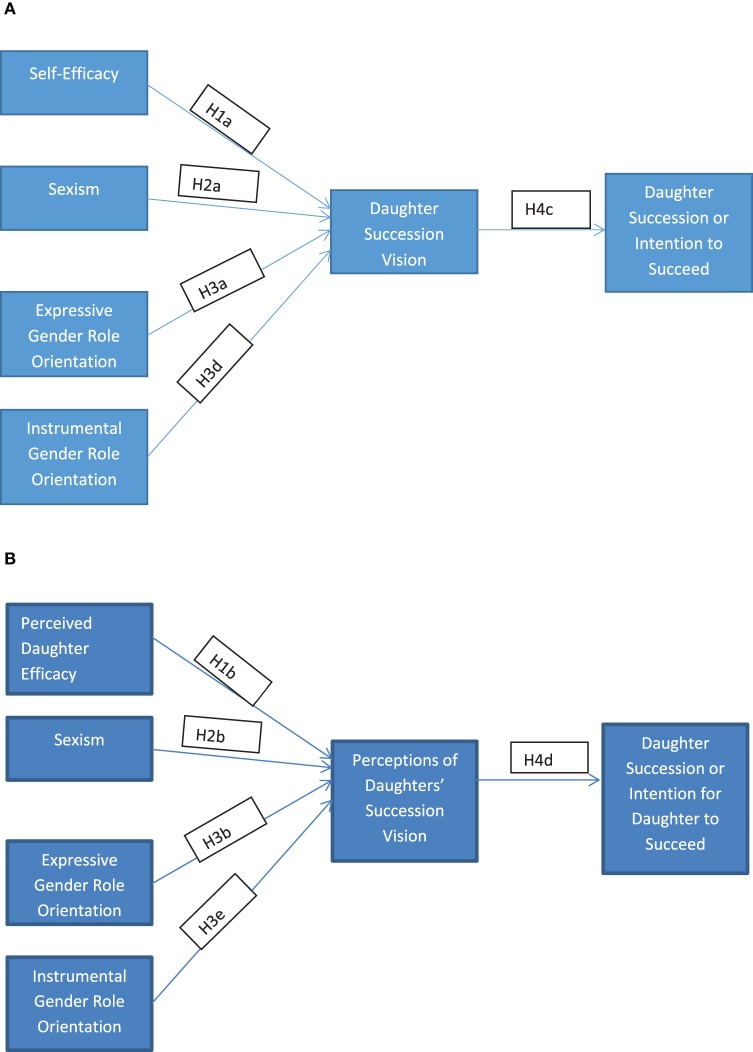
**Hypothesized models (A,B). (A)** Hypothesized daughter model. **(B)** Hypothesized fathers' model.

In the first hypothesized model shown in Figure 1A, we propose that self-efficacy, sexism, and expressive and instrumental gender role orientations, are contributors to daughter succession through the mediator, Daughter Succession Vision. Since fathers are typically gatekeepers to succession, we further compare fathers' perceptions of daughters as family business leaders or successors. Accordingly, in Figure 1B, we hypothesize that perceived daughter efficacy (fathers' perceptions of daughters' efficacy), sexism, and expressive and instrumental gender role orientations influence perceived daughter vision (fathers' perceptions of daughters' succession vision, i.e., mediator), which in turn influences the outcome of the vision (i.e., dependent variable–daughter succession). Next, we discuss the key constructs and develop our hypotheses.

### The roles of self–efficacy and perceived daughter efficacy

Social cognitive theory proposes that personal evaluative processes are the foundations of human agency. Unless people believe they can achieve desired goals, they have little incentive to exert necessary effort or persevere in the face of difficulties (Bandura, 2001). Efficacy beliefs influence aspirations, choices, vulnerability to stress and depression, and performance accomplishments (Fernandez-Ballesteros et al., 2002).

According to social cognitive theory, self-efficacy refers to a self-evaluative process that links reasoning to conduct (Bandura, 2001). It is a judgment of capabilities to execute activities required to meet desired goals. In the present study, self-efficacy refers to a daughter's self-reported evaluation of her ability to achieve success as a leader in the family business. It is domain specific (Bandura, 2006a,b) since a general sense of self-efficacy may have little or no relevance to functioning in the family business (DeNoble et al., 2007). For example, a daughter may have strong beliefs in her ability to achieve in school, sports, or social causes, but may not believe she can lead the family business.

Similarly, fathers' judgments of daughters' efficacy may inform choices and link reasoning to conduct. As leaders of the family business and selectors of successors, fathers' assessments of daughters' capabilities to execute tasks to achieve desired goals may influence fathers' decisions to appoint daughters as successors. The construct, “perceived daughter efficacy,” in our model reflects fathers' self-reported perceptions of daughters' efficacy as leaders of family businesses.

We examine the effects of self-efficacy and perceived daughter efficacy in two ways. First, we assess levels of beliefs about daughters' capabilities to lead the family business. In both models, strong beliefs about daughters' capabilities to lead the family business will influence daughters' dreams or vision for the future of the business and succession outcome, or daughters' ascendency to the position of successor. Secondly, we examine the variability in beliefs about daughters' capabilities between members of each daughter/father pair. We hypothesize that the closer the beliefs of the members of the family business pair, the stronger the predictability of the outcome. If fathers and daughters agree that daughters would not be efficacious leaders of the business, daughters will not likely develop a view for the company's future or achieve successor status. Contrastingly, if fathers and daughters agree that daughters have the ability to lead the company profitably, it is likely daughters will develop dreams or visions for the future of the company and succession.

We predict that a comparison between fathers' and daughters' perceptions of daughters' efficacy will differ. Previous studies on daughters in family businesses have suggested that daughters are usually invisible to fathers as potential leaders in family businesses (Curimbaba, 2002; Jimenez, 2009). Gender biases are noted to contribute to invisibility (Hollander and Bukowitz, 1990; Dumas, 1992; Lozano et al., 2011; Overbeke et al., 2013). Therefore, it is likely that more daughters will perceive they have successor efficacy than will their fathers.

We hypothesize:

*Hypothesis 1a. Daughters' self-efficacy is positively associated with Daughter Succession Vision in family businesses*.
*Hypothesis 1b. Fathers' perceptions of daughters' efficacy is positively associated with Perceived Daughter Vision in family businesses*.
*Hypothesis 1c. Fathers and daughters beliefs about daughters' efficacy are significantly different, with daughters believing they have stronger levels of efficacy*.

### The role of sexism

Sexism refers to discriminatory practices against women, including overt and nuanced or subtle forms of sexism (Benokraitis and Feagin, 1986; Benokraitis, 1997; Jandeska and Kraimer, 2005). Overt sexism, or acts such as sexual harassment that can be documented or easily distinguished, have become less socially acceptable. Yet, women continue to experience gender based discriminatory behaviors and practices. Sexism may, therefore, be subtle or clandestine (Benokraitis and Feagin, 1986; Benokraitis, 1997; Swim and Cohen, 1997) which are not as detectable, but powerful. These acts of sexism are embedded in cultural and societal norms and permeate multiple levels of society, including individual, organizational, institutional, and cultural. Covert sexism influences education, politics, religion, law, and other environmental factors (Benokraitis, 1997) that may influence daughter succession.

While both overt and covert sexism may be experienced by daughters of family businessmen, this research project focuses on subtle or covert sexism. Covert sexism is perhaps more ubiquitous and less understood in the context of daughters and succession. Many fathers and daughters may not be aware of embedded norms and how they may restrict a vision for the future of the family business with daughters at the helm. Benokraitis (1997) theorized that there are nine forms of subtle sexism: condescending chivalry, supportive discouragement, friendly harassment, subjective identification, radiant devaluation, liberated sexism, benevolent exploitation, considerate domination, and collegial exclusion. The names of these categories are oxymorons, meant to highlight the mixed messages they send and to emphasize the seemingly supportive behavior that has pernicious consequences (Benokraitis, 1997).

Three illustrations help explain how subtle sexism is exercised and its impact on family businesses. Examples of supportive discouragement, liberated sexism, and collegial exclusion show contradicting messages delivered through socially accepted actions. These mechanisms may discourage daughters from becoming successors. Supportive discouragement refers to a form of subtle sex discrimination where women are encouraged to succeed, in general, but numerous obstacles are placed in their path, intended to limit or derail their progress. Benokraitis offers the example of a college that offered a part-time program for people with no formal qualifications who wish to enter or reenter the workforce. Most participants were women with young children and required child care while in class or studying. The college also had a department dedicated to training child care workers and the two departments appeared to be a natural match. Students learning to be childcare workers could benefit from providing child care for the part-time mothers. However, despite the availability of a volunteer or low cost staff, the college refused to offer child care for the part-time students. In addition, the college housed the part-time program in the worst building on campus, thus appearing to offer women an opportunity but discouraging them with inadequate support.

Similarly, parents may use verbal persuasion to instill a sense of competency in their daughters. Daughters may be told they may aspire to anything, yet a structure of opportunities leading to succession typically excludes daughters (Iannarelli, 1992; Curimbaba, 2002). They are not encouraged to follow an educational curriculum preparing them for business and daughters who enter the family business are often not included in important decision-making or discussions (Barnes, 1988; Hollander and Bukowitz, 1990; Dumas, 1992). Furthermore, female management styles that differ from males often cause fathers to conclude that daughters are ineffective leaders (Dumas, 1992). The more collaborative and caring style of women managers are often misconstrued as uncompetitive and damaging to business. Thus, daughters are verbally, but not materially supported.

Liberated sexism refers to a process where equality is presumed, but in practice, men's freedom increases while women's workload increases. The best example is that of employed mothers of pre-school aged children. Most spend an average of 24 h or more per week on child care activities than their husbands (Benokraitis, 1997). These “liberated” women therefore have two jobs—one in the home and one outside the home. Successor daughters may also be expected to be the primary caretaker of her children while working outside the home. Additionally, liberated sexism has a unique twist in family businesses. Fathers represent both the company and family; consequently, they often expect devotion to the firm and simultaneously ask, “When are you going to give me a grandchild?” (Cole, 1997: 16).

Collegial exclusion refers to a form of subtle sexism where women are made to feel invisible or unimportant through physical, social, or professional isolation. Many women in non-traditional jobs find themselves being ignored, without role models, or excluded from discussions and social activities. Benokraitis (1997) relays an anecdote about a female college president meeting with other college presidents. As the only female among them, she found her suggestions and insights were ignored. However, when the same suggestions were later offered by a male colleague, they were acknowledged. Collegial exclusion has unique implications for family businesses because grooming of successors often begins at an early age (Dumas, 1992) where explicit and tacit knowledge are transferred from predecessor to successor through activities and conversation (Cabrera-Suarez et al., 2001). Daughters usually do not expect to become successors (Jimenez, 2009) and do not share in the grooming process. Collegial exclusion thus begins at an early age in family businesses.

Benokraitis began researching subtle sexism in the 1990s, but there is evidence of the persistence of this practice into the 21st century. The discrepancy between the number of highly trained professional women and the number of women in the highest executive ranks offers evidence of subtle sexism (Jandeska and Kraimer, 2005). Women are hired into management positions with ostensibly the same opportunities as their male colleagues to advance in an organization. Unofficial institutions such as the “masculine code of conduct” and “old boys networks” create an exclusionary culture that prevents access to information and opportunities for dialog. This is a form of collegial exclusion that causes women to become demoralized and less committed to an organization (Jandeska and Kraimer, 2005).

Daughters of family businessmen may also experience an opportunity gap created by an exclusionary climate toward women. The impact of this form of subtle sexism may be stronger because of the juxtaposition of the family and business. If found in the home, family, and business, it is unlikely daughters would have an interest in the business. They would be discouraged from developing a view of the future for the business, a dream that would precede leadership action. It follows that fathers who are not perceptive of subtle forms of sexism will not recognize these obstructions to daughters' succession. Sexism is therefore an attenuating variable with a restricting impact on vision and succession. We posit:

*Hypothesis 2a. Daughters' beliefs that sexism is strong in American society has a strong impact on whether or not daughters form a Daughter Succession Vision*.
*Hypothesis 2b. When fathers' subtle or covert sexist beliefs and attitudes increase, the likelihood they perceive daughters have a succession vision decreases*.
*Hypothesis 2c. Daughters believe there is more sexism in society than do fathers*.

### The role of gender role orientation

Gender role orientation refers to beliefs about the proper roles for men and women at work and in the home (Judge and Livingston, 2008). Early theorists believed that gender was inborn, (Spence and Buckner, 2000) and characterized men as “instrumental” and women as “expressive.” Instrumentality meant that men are predisposed to “get things done” and are, therefore, more qualified for managing economic and political institutions. Conversely, expressiveness represents caring, nurturing, and other qualities that are better suited for domestic responsibilities (Whitley, 1983; Spence and Buckner, 2000; Judge and Livingston, 2008; Mueller and Dato-On, 2008). Implicit in Expressive Gender Role Orientation is the subordination of women and their need for protection (Spence and Buckner, 2000; Mueller and Dato-On, 2008).

Early theories of gender role orientation were based on the assumption that “masculinity and femininity are opposite poles of a single dimension.” That is, one must have either a masculine or a feminine sex-role orientation, “because these role orientations are mutually exclusive and incompatible” (Whitley, 1983: 766). This unidimensional understanding of gender role orientation was challenged in 1973 by Constantinople who developed a framework positing male and female sex roles as independent constructs (Mueller and Dato-On, 2008). This conceptualization led to non-traditional perspectives of gender as socially constructed rather than biologically determined sex roles (Mueller and Dato-On, 2008).

More recent theorists have built on Constantinople's conceptualization, proposing that gender role orientation is determined by individual attitudes, values, and self-concepts. Consequently, expressiveness may manifest in males and instrumentality may be exhibited by females (Spence and Buckner, 2000; Judge and Livingston, 2008; Mueller and Dato-On, 2008). In the present study, gender role orientation is seen as the operationalization of how strongly daughters are seen as expressive or instrumental and how that influences the selection and self-selection of a successor. Hackett and Betz's work (1981) established a link between gender beliefs and career choices. We measure the extent to which fathers' and daughters' beliefs about appropriate occupations for daughters are driven by beliefs about appropriate roles for men and women.

Several recent studies suggest that the social environment in the United States has become more egalitarian (Spence, 1993; Mueller and Dato-On, 2008), but signs of traditional views of the division of labor among men and women endure (Jandeska and Kraimer, 2005). Most prominent among these signs is a persistent gender wage gap which can be linked to gender role orientation (Judge and Livingston, 2008). In a longitudinal study, Judge and Livingston found a strong positive correlation between traditional gender role orientation and earnings for men and a slightly negative correlation with earnings for women. Similar to Hackett and Betz's (1981) findings, Judge and Livingston (2008) explain that gender role socialization leads individuals to find jobs dominated by their own gender. They argue that women with more traditional gender role orientations experience cognitive dissonance or discomfort when working in jobs usually held by men.

As a predictor of succession in family businesses, gender role orientation evaluates socialization factors, cultural conditioning, and cognitive perceptions of gender appropriate occupations. The more traditional a daughter's gender role orientation, the less likely she will become a successor. The more traditional a father's gender role orientation, the less likely he will appoint her successor. Finally, if the father and daughter have significantly different gender role orientations, and the father's is more traditional, the daughter will not likely be a successor. We therefore hypothesize:

*Hypothesis 3a. There is a negative association between Expressive Gender Role Orientation and Daughter Succession Vision as perceived by daughters*.
*Hypothesis 3b. There is a negative association between Expressive Gender Role Orientation- and Daughter Succession Vision as perceived by fathers*.
*Hypothesis 3c. Fathers' and daughters' perceptions of daughters' Expressive Gender Role Orientation are significantly different, with daughters believing they have lower levels of expressiveness*.
*Hypothesis 3d. There is a positive association between Instrumental Gender Role Orientation and Daughter Succession Vision as perceived by daughters*.
*Hypothesis 3e. There is a positive association between Instrumental Gender Role Orientation and Daughter Succession Vision as perceived by fathers*.
*Hypothesis 3f. Fathers' and daughters' beliefs about daughters' Instrumental Gender Role Orientation are significantly different, with daughters believing they have higher levels of Instrumental Gender Role Orientation*.

### Daughter succession vision

Drawing on Intentional Change Theory's (ICT) conceptualization of personal vision (Boyatzis, 2006), Daughter Succession Vision represents a view of desired leadership needed to achieve a desired future of the family business. At an individual level it is an aggregated image including an assessment of the Ideal Leader compared to the Real Leader. At the collective level it is a shared vision (Boyatzis and Akrivou, 2006) of hopes and dreams for the future of the family business between fathers and daughters.

In ICT, personal vision is a consequence of the Ideal Self. The Ideal Self “is an evolving, motivational core within the self, focusing a person's desires and hope, aspirations and dreams, purpose and calling” (Boyatzis, 2006: 625). It leads to a manifestation of an image of what kind of person one wishes to be, or a personal vision (Boyatzis and Akrivou, 2006). In comparison, the Real Self in ICT is an examination of one's current self, the person that others see along with an internal assessment of personal beliefs and emotions. It includes an exploration of questions such as, “Who am I?” (Boyatzis, 2006) “How am I fitting into this setting? How am I doing in the view of others? Am I part of this group or organization or family?” (Boyatzis, 2006: 15).

The “Ideal Leader” relates to summary judgments of qualities necessary for a leader of the family business. Like the Ideal Self, the Ideal Leader encompasses a focus on hope, dreams and aspirations, and purpose and calling within the family business. Just as the “Real Self” is an assessment of the person that others see, the “Real Leader” is an assessment of the person that others see in the context of leadership in the family business.

A comparison of the Ideal Leader with the Real Leader allows an evaluation of a successor candidate's fit with the image of the ideal leader. In this study, the daughter represents the “Real Leader” and is compared with her own appraisal of the Ideal Leader. Additionally, the father compares the Real Leader, or the daughter, with his image of the Ideal Leader and this leads to a vision of a successor.

The Real Leader is supported by two dimensions, motivation and readiness. Similar to the construct of intention (Ajzen and Driver, 1991), these components suggest action. Motivation represents the desire to advance the effort required to become a successor. For example, if a daughter is not highly motivated to be a successor, she will not pursue this position, despite other factors. Hence, motivation is considered when constructing an image of a successor and is a criterion when selecting a successor. Likewise, readiness suggests actions taken to prepare for succession. As an indicator of a Real Leader, it implies the amount of effort already extended toward succession. Examples include reading books, taking business courses, or participating in the family business while growing up.

In the present study, Daughter Succession Vision will be assessed individually and comparatively on two levels. First, the father and daughter's individual visions will be determined. What qualities does the father think are necessary in a successor in order to achieve his view of the future of the family business? How does his daughter compare with his vision of a successor? How does the daughter perceive these same issues? Secondly, how do fathers' and daughters' visions compare with each other? How does the father's vision of his daughter as a successor compare with the daughter's assessment of herself as a successor?

In sum, Daughter Succession Vision is a complex variable that collects and disseminates information. Daughter Succession Vision accumulates various factors into a comprehensive variable, a desired future. This predicts the outcome through an interactive process between the Ideal Leader and Real Leader. The level of strength with which the daughter fits the image of the Ideal Leader determines the likelihood the father will choose his daughter to become a successor and the likelihood the daughter self-selects as a successor. Differences between levels of strength may reveal gender biases. Fathers may be blind to daughters' visions because they do not perceive their daughters to be capable of leading family businesses. We therefore propose:

*Hypothesis 4. Daughters' “Daughter Succession Visions” are stronger than fathers perceive*.

### Mediation effects

Daughter Succession Vision/Perceptions of Daughters' Succession Vision are positioned as mediators in our models. As described by Mathieu and Taylor (2006), mediators “elucidate the underlying mechanisms linking antecedents and their consequences” (p. 1031). Thus, mediators are not merely linking variables but provide theoretical understanding of the connection between independent and dependent variables. As mediators, Daughter Succession Vision/Perceptions of Daughters' Succession Vision are expected to reduce the direct effects of the independent variables on the dependent variable. Self-Efficacy/Perceived Daughter Efficacy, beliefs about sexism, and gender role orientations, are predicted to have direct relationships with succession outcome. The aggregated effects of these variables, however, may be explained by Daughter Succession Vision/Perceptions of Daughters' Succession Vision. For example, a father may believe his daughter can execute functions leading to a successful business. Hence, a direct relationship may exist between Successor Efficacy/Perceived Daughter Efficacy and Daughter Succession. Yet, this father may also believe it is inappropriate for women to manage a business. Daughter Succession is therefore explained by the father's perceptions or assumptions about whether their daughter has a vision for the future of the family business.

*Hypothesis 4a. Daughter Succession Vision mediates the effects of daughters' assessments of: (1) Self-Efficacy, (2) Sexism, (3) Expressive Gender Role Orientation, and (4) Instrumental Gender Role Orientation, on Daughter Succession*.
*Hypothesis 4b. Perceptions of Daughters' Succession Vision mediates the effects of fathers' assessments of: (1) Perceived Daughter Efficacy, (2) Sexism, (3) Expressive Gender Role Orientation, and (4) Instrumental Gender Role Orientation, on Daughter Succession*.
*Hypothesis 4c. The stronger Daughter's Succession Visions, the more likely daughters will associate with Daughter Succession*.
*Hypothesis 4d. Fathers who perceive daughters have strong Daughter Succession Visions will positively associate daughters with Daughter Succession*.

## Methods

### Research setting and sampling procedures

The target population for this study was pairs of fathers and daughters where fathers owned a family business and daughters were over the age of 18. A family business was defined as a business where the families have control over the business' strategic direction and there is some family participation in the business (Astrachan and Shanker, 2003). Eight associations with family business memberships ranging from 15 to 17,000 were contacted. These associations were selected based on the size and diversity of its business population. Three associations agreed to participate in the collection of data: (1) a branch of the Cleveland Ohio Chamber of Commerce; (2) a university based family business organization; and (3) a national professional association.

In addition, the difficult nature of data collection for this project finally required a convenience sample including a “snowball” method of collection. In total, researchers sent a mass email with links to the surveys to 228 individuals, including 133 fathers and 95 daughters. Initial emails were followed by reminders until 50 pairs responded. Each father/daughter pair were asked to choose a unique identifying name so that their surveys could be paired and they could retain anonymity. A summary of completed demographic questions may be seen in Table 1.

**Table 1:** **Demographic profiles of respondents and organizations**.

**Daughters and succession**	**Number**	**Percent**
Daughters currently working in family business	23	46
Daughters currently in successor positions	7	14
Daughters with high intentions of succession	10	20
Daughters with low intentions of succession	18	36
Daughters undecided	15	30
**INDUSTRIES**		
Service	15	30
Wholesale	2	4
Manufacturing	1	2
Unreported	32	64
**GROSS REVENUES**		
Less than $100,000	2	4
$100,000–499,999	8	16
$500,000–999,999	1	2
$1,000,0000–4,999,999	18	36
$5,000,000–9,999,999	5	10
$10,000,000–49,999,999	8	16
$50,000,000–99,999,999	2	4
Over $100,000,000	6	12
**GENERATION CURRENTLY OPERATING BUSINESS**		
1st	24	48
2nd	16	32
3rd	8	16
4th	1	2
More than 4th	1	2
**AGE-FATHERS**		
50–53	8	16
54–58	15	30
59–61	10	20
62–64	8	16
65–67	6	12
No response	3	6
**AGE-DAUGHTERS**		
19–28	26	52
29–38	18	36
39–48	2	4
49–53	0	
54–58	4	8

### Data collection

The survey instrument was pre-tested by two-panels for face validity and appropriate interpretation of questions. The panels were composed of academic researchers, fathers owning family businesses whose daughters were too young to participate, and daughters of family businessmen who did not qualify to participate in the study (i.e., daughters whose fathers were deceased). Those participating in the pre-tests examined question structure and order, item consistency, and clarity of construct dimensions. Critical review resulted in some revisions and further honing of the survey instrument so that questions were less ambiguous and response choices made sense (Dooley and Lindner, 2003). Verbal labels, clarifying the meaning of scale points (Krosnick, 1999) were also examined for clarity.

### Measurement

Two surveys, with separate links, were hosted by an online survey company. The focal object of both surveys was the daughter. Fathers were asked to evaluate their daughters and daughters were asked to evaluate themselves. Fathers' and daughters' surveys were identical except for necessary word changes to direct respondents' attention to the daughter.

Previously validated scales, chosen for their theoretical and empirical properties, were used to measure constructs. Some scales were modified to contextualize the items to reflect assessments within a family business. For example, the “New General Self-Efficacy Scale” (Chen et al., 2001) was used to measure daughters' self-efficacy and fathers' perceptions of daughters' efficacy. Table 2 shows the first item in the original scale and how it was adapted or contextualized to fit daughters' and fathers' surveys:

**Table 2:** **Example of contextualized self-efficacy scale item**.

**New general self-efficacy scale (Chen et al., 2001)**	**Present daughter survey**	**Present father survey**
1. I will be able to achieve most of the goals that I have set for myself	1. I will be able to achieve most of the goals that I set for myself as an executive in my family's business.	1. My daughter will be able to achieve most of the goals that she sets for herself as an executive in our family business.

All measurements, with the exception of the dependent variable, Succession, were based on a 5-point Likert scale with 1 = strongly disagree and 5 = strongly agree. Details about all scales employed are provided in Appendix A.

### Dependent variable

The dependent variable, daughter succession, was defined broadly in order to include both daughters who were on a path toward succession and daughters who were already in an executive leadership position. This study measures fathers' and daughters' perceptions of the desired qualities of family business successors, how well daughters match those qualities, and the influence of gender bias on these perceptions. Unfortunately, the sample size of daughter successors was so small it was necessary to combine it with daughters intending to become successors. Using this definition of daughter succession, the dependent variable, Daughter Succession, was calculated by adding the codes assigned to two scales. Intention was measured with a 5-point Likert scale and the Daughter Succession scale was measured by assigning codes to the daughter's reported position title within the family business. The codes assigned to daughter's position title are reported in Table 3.

**Table 3:** **Codes assigned to daughter's current position title**.

**Rank**	**Position title**
7	CEO
	COO
	President
6	Vice-president
5	Director
4	Manager
3	Technical
	Sales person
	Coordinator
2	Administrative
1	Not in the family business

Items measuring Intention are described in Appendix A. The highest possible score combining rank and intention was 12. We considered scores higher than half of 12 to be indicators of succession or future succession.

### Gender role orientation

A modified version (Valian, 1998) of the Personal Attributes Questionnaire (PAQ) (Spence and Helmreich, 1978; Spence and Buckner, 2000) was used to measure gender role orientation. Currently, there are several scales available to measure gender role attitudes. The PAQ has been noted for containing the least social desirability bias (Whitley, 1983). The PAQ is widely used for measuring instrumental and expressive personality traits that are stereotypically associated with men and women (Fernandez et al., 2007). The scale consists of 24 items measured on a five point Likert scale. There are two dimensions in this scale, each consisting of eight items: (1) instrumental (α = 0.77) and, (2) expressive (α = 0.51). The remaining eight items are “fillers” for reducing bias (Spence, 1993: 628).

### Sexism

The Modern Sexism Scale (Swim and Cohen, 1997) was used to assess fathers' and daughters' perceptions of discriminatory practices against women in American society. This scale consists of eight items measured on a 5-point Likert scale and measures both overt and subtle forms of sexism (Benokraitis and Feagin, 1986).

### Self-efficacy/perceived daughter efficacy

“The General Self-Efficacy Scale,” (α = 0.81; Chen et al., 2001) employs eight items measuring perceptions of skills and abilities to successfully perform tasks in a variety of settings. This scale was contextualized to assess a daughter's self-efficacy and her father's perception of collective efficacy. Thus, the father's scale measures his perceptions of the efficacy of the business organization with his daughter at the helm.

### Daughter succession vision

“The Personal Vision” scale, taken from the PNEA Survey (α = 0.92; Boyatzis and Oliver, unpublished) consists of eight items based on a 5-point Likert scale. The scale measures the extent to which a daughter and her father view the daughter as a successor in the family business.

### Intention

This construct was measured using a four item scale adapted from Lin (2007). The scale was adapted to a 5-point Likert scale from a 7-point Likert scale.

### Method of analysis

The research model was tested using Partial Least Squares (PLS-Graph, v3.0, Build 1060, Chin and Frye, 1998). The hypothesized relationships among constructs were analyzed using the partial least squares (PLS) approach for structural equation modeling (SEM). The decision to use PLS, rather than a covariance-based SEM (supported by such tools as LISREL and AMOS), was based primarily on the goal and nature of the study. The study's aim was to understand how well the model predicts daughter's succession, rather than to explain covariance of all measures. The study is based on a concept that has not been explored and is little understood. The nature of modeling succession lends itself to an exploratory data analysis approach. Prediction—rather than explanation—orientation of the study, as well as the lack of a strong theory, makes PLS a very suitable parameter estimation methodology (Chin, 1998; Haenlein and Kaplan, 2004).

Prior to hypotheses testing, data were screened for missing cases and checked for regression assumptions of homoscedasticity, multicollinearity, and linearity. The correlation matrix, means, and standard deviations are presented in Table 4.

**Table 4:** **Descriptive statistics and construct correlations**.

	**Mean**	**S.D**.	**IGRO**	**EGRO**	**SEFF**	**VIS**	**INT**	**SX**	**DS**
IGRO	3.74	0.72	**0.75**						
EGRO	4.1017	0.65	0.200^*^	**0.75**					
SEFF	4.12	0.68	0.671^**^	0.274^**^	**0.84**				
VIS	3.32	0.84	0.317^**^	0.143	0.511^**^	**0.81**			
INT	3.01	1.21	0.125	−0.097	0.228^*^	0.606^**^	**0.92**		
SX	2.61	0.73	0.140	−0.022	0.097	0.188	0.216^*^	**0.77**	
DS	5.25	2.64	0.143	−0.135	0.201^*^	0.529^**^	0.856^**^	0.147	**n/a**

* *Correlation is significant at the 0.05 level (2-tailed)*.

** *Correlation is significant at the 0.01 level (2-tailed)*.

### Common method bias (CMB)

The data collection instrument for this study was a self-report survey. Such a format often lends itself to method bias. We tested for method bias by examining correlations among latent variables (see Table 1). All correlation values are far below the suggested maximum threshold of 0.90 (Pavlou et al., 2007). Next, we conducted a Harman's single factor test wherein all variables are loaded onto one factor while conducting a principal components factor analysis. According to this test, if one factor emerges explaining over 50% of the model, CMV is determined to be present (Podsakoff et al., 2003). Results indicated one factor explaining 24.7% of the model, suggesting that CMV is present, but not strong enough to produce a significant bias.

Psychometric properties of the EFA model were examined for construct reliability and convergent validity. Exploratory Factor Analysis (EFA) was conducted in SPSS using Principal Axis Factoring and Promax rotation. The reliability of the scale items was assessed for internal consistency using Cronbach's alpha. The initial 42 items yielded a five factor solution and items loaded as hypothesized, explaining 59% of the variance in the model. Items with low loadings or cross loadings were removed. After several iterations, a trimmed model presented 34 items explaining 54% of the model variance. The constructs' Cronbach's alpha measurement exceeded 0.75, indicating internal consistency among survey responses.

Next, the factorial validity of the measured constructs was evaluated with a confirmatory factor analysis model. We examined factor loadings, composite reliability (CR), and average variance extracted (AVE) measures. Both CR (0 ≤ CR ≤ 1) and AVE (0 ≤ AVE ≤ 1) are commonly used metrics of convergent validity (Hair et al., 2010). Both CR and AVE metrics exceeded the acceptable thresholds of 0.7 and 0.5, respectively (Hair et al., 2010), providing evidence of construct reliability and convergent validity. Further, the analysis assessed discriminant validity using AVE and inter-factor correlations in combination. Discriminant validity can be established if a latent variable's AVE is larger than the common variances (Chin, 1998; Pavlou et al., 2007; Götz et al., 2010). Following this guidance, Table 4 presents the square root of AVE for each construct on the diagonal (in bold) to compare against the correlations among the constructs captured in the off-diagonal elements of the matrix. Table 5 shows that all constructs demonstrate both CR and discriminant validity.

**Table 5:** **Factor loadings and measurement properties of construct**.

**Construct/Item**	**Loading**	***t*-value**	**Composite reliability**
**INSTRUMENTAL GENDER ROLE ORIENTATION**			
Q1_7	0.61	5.23	0.863
Q1_10	0.75	10.27	
Q1_11	0.76	7.73	
Q1_12	0.74	5.49	
Q1_15	0.86	23.36	
**EXPRESSIVE GENDER ROLE ORIENTATION**			
Q1_4	0.73	4.59	0.881
Q1_5	0.53	2.63	
Q1_6	0.91	6.79	
Q1_8	0.84	5.56	
Q1_13	0.74	4.61	
Q1_14	0.68	3.61	
**SELF-EFFICACY/PERCEIVED DAUGHTER EFFICACY**			
Q2_1	0.83	22.02	0.948
Q2_2	0.86	29.77	
Q2_3	0.85	28.91	
Q2_4	0.85	20.25	
Q2_5	0.87	30.89	
Q2_6	0.88	29.49	
Q2_7	0.72	10.99	
Q2_8	0.81	15.76	
**SEXISM**			
Q10_2	0.87	8.16	0.90
Q10_3	0.58	3.29	
Q10_6	0.80	5.38	
Q10_7	0.81	6.60	
**DAUGHTER SUCCESSOR VISION/PERCEPTIONS OF DAUGHTER SUCCESSION VISION**			
Q3_1	0.80	20.70	0.92
Q3_2	0.84	29.72	
Q3_3	0.79	18.78	
Q3_4	0.74	9.31	
Q3_5	0.84	23.80	
Q3_6	0.84	25.33	

## Analysis and findings

Testing of the structural model was conducted in two stages. First, path relationships were tested using a bootstrapping procedure in PLS. Secondly, a matched pair *T*-Test was conducted in SPSS in a post hoc analysis, to determine significant differences between fathers' and daughters' responses across all scale items. The hypothesized structural model was examined in PLS using two separate data sets, one reflecting fathers' responses and the other composed of daughters' responses. Two models were created, as the data suggested different findings for the father and daughter groups. We will first present findings from the path analyses between independent variables and the mediator. An examination of mediated relationships will follow. Finally, we present a comparative analysis between fathers' and daughters' responses.

Regression weights and corresponding significance levels for each hypothesized construct relationship in the father's model indicated two paths between independent variables and the mediator were significant and two paths were not as posited. The supported hypotheses in the fathers' model were:

*Hypothesis 1b. Fathers' perceptions of daughters' successor efficacy is positively associated with Daughter Succession Vision in family businesses*.
*Hypothesis 2b. When fathers' beliefs about women in society reflect subtle or covert sexist attitudes, they will not perceive that daughters have a Daughter Succession Vision*.

Rejected hypotheses in the fathers' model were:

*Hypothesis 3b. There is a negative association between Expressive Gender Role Orientation and Perceptions of Daughters' Succession Vision*.
*Hypothesis 3e. There is a positive association between Instrumental Gender Role Orientation and Perceptions of Daughters' Succession Vision*.

In the Daughter's model two paths between independent variables and the mediator were significant and two paths were not as predicted. The supported hypotheses in the daughters' model were:

*Hypothesis 1a. Daughters' perceptions of self- efficacy are positively associated with Daughter Succession Vision in family businesses*.
*Hypothesis 2a. A belief that sexism is strong in American society has a strong impact on whether or not daughters form a Daughter Succession Vision*.

The rejected hypotheses in the daughters' model were:

*Hypothesis 3a. There is a negative association between Expressive Gender Role Orientation and Daughter Succession Vision as perceived by daughters*.
*Hypothesis 3d. There is a positive association between Instrumental Gender Role Orientation and Daughter Succession Vision as perceived by daughters*.

Table 6 summarizes the hypotheses, showing coefficients and significance levels for proposed relationships between Independent Variables and the Mediator, and the relationship between the Mediator and the Dependent Variable. For clarity, estimates and significance levels of mediated relationships are reported in a separate table.

**Table 6:** **Summary of hypothesis testing**.

**Hypothesis**	**Path/Respondent [Daughter (D)or Father (F)]**	**Hypothesized model coefficient**	***t*-value**	**Hypothesis supported**
H1a	Successor efficacy→Vision-D	0.387	2.33	yes
H1b	Successor efficacy→Vision-F	0.435	2.09	yes
H2a	Expressive gender role orientation→Vision D	0.073	0.78	no
H2b	Expressive gender role orientation→Vision F	0.045	0.43	no
H2d	Instrumental gender role orientation→Vision D	0.080	0.60	no
H2e	Instrumental gender role orientation→Vision F	−0.025	0.16	no
H3a	Sexism→Vision D	0.377	3.40	yes
H3b	Sexism→Vision F	0.218	1.94	yes
H4c	Vision→Succession or intention to succeed-D	0.542	6.00	yes
H4d	Vision→Succession or intention to succeed-F	0.598	6.45	yes

*D = Daughters; F = Fathers*.

### Mediation analysis

While mediator variables explain the nature of a relationship between two variables (X→M→Y), a relationship between X→Y must be established before a predictor and criterion may be evaluated for mediation (Mathieu and Taylor, 2006). The X→Y precondition is examined in Table 7.

**Table 7:** **Mediation preconditions**.

**Mediation**	**Daughters**				**Mediation**	**Fathers**			
**X→Y**	**Coefficient**	***t*-value**	**St. error**	**Sig**.	**X→Y**	**Coefficient**	***t*-value**	**St. error**	**Sig**
EGRO→DV	−0.1210	0.9501	0.1274	no	EGRO→DV	−0.2560	2.1170	0.1209	Yes^*^
IGRO→DV	0.0600	0.4907	0.1223	no	IGRO→DV	−0.1530	1.1804	0.1296	no
SEFF→DV	0.2380	1.4966	0.1590	no	SEFF→DV	0.130	1.0690	0.1244	no
SX→DV	0.2520	2.2505	0.1120	Yes^*^	SX→DV	0.0430	0.4168	0.1032	No

EGRO, Expressive Gender Role Orientation; IGRO, Instrumental Gender Role Orientation; SEFF, Successor Efficacy; SX, Sexism.

* *Significant at 0.05 level*.

The precondition tests showed a significant relationship between Sexism and Daughter Succession in the daughters' model. It also revealed a significant relationship between Expressive Gender Role Orientation (EGRO) and Daughter Succession in the fathers' model. Other variables were not significantly related to the DV. These tests suggest that further mediation testing may indicate mediation, partial mediation, and unanticipated effects such as direct or indirect effects.

Full and partial mediation results are presented in Tables 8, 9.

**Table 8:** **Mediation-daughters**.

**Path**	**Coefficient**	***T*-value**	**Standard error**	**Significance**
Sexism→Vision	0.3190	2.4790	0.1287	yes
Vision→Succession	0.4860	4.2413	0.1146	yes
Sexism→Succession	0.1230	1.2017	0.1024	no

**Table 9:** **Mediation-fathers**.

**Path**	**Coefficient**	***T*-value**	**Standard error**	**Significance**
Expressive gender role orientation→Vision	−0.0070	0.0705	0.0993	no
Vision→Succession	0.6350	6.0041	0.1058	yes
Sexism→Succession	−0.2560	2.1170	0.1209	yes

Tables 8, 9 show full mediation in the Daughters' model and partial mediation in the Fathers' model. In the Daughters' model paths between the IV (Sexism) and Mediator (VIS), and the Mediator (VIS) to the DV (Daughter Succession) are significant. The path between the IV and DV is not significant, suggesting that Daughter Succession Vision is necessary to explain the relationship between Sexism and Daughter Succession.

In the Fathers' model paths between the IV (EGRO) and DV (Daughter Succession), and the Mediator (VIS) and DV (Daughter Succession) are significant. The path from the IV (EGRO) to the Mediator (VIS) is not significant. This suggests partial mediation as EGRO has a direct relationship with the DV, but may be influenced by the mediator, VIS. Table 10 presents direct and indirect effects of VIS.

**Table 10:** **Daughters-mediated, direct and indirect effects/fathers-direct and indirect effects**.

**Path**	**Coef**	***t*-value**	**St. err**	**Sig**	**Effect**
**DAUGHTERS-MEDIATED, DIRECT AND INDIRECT EFFECTS**					
EGRO→VIS	0.1210	1.2636	0.0958	NO	NONE
VIS→DV	0.4860	4.2413	0.1146	YES	
EGRO→DV	−0.2050	1.5227	0.1346	NO	
IGRO→VIS	0.1080	0.7692	0.1404	NO	NONE
VIS→DV	0.4860	4.2413	0.1146	YES	
IGRO→DV	−0.0400	0.2741	0.1460	NO	
SX→VIS	0.3190	2.4790	0.1287	YES	MED
VIS→DV	0.4860	4.2413	0.1146	YES	
SX→DV	0.1230	1.2017	0.1024	NO	
SEFF→VIS	0.3880	2.0212	0.1920	YES	INDIRECT
VIS→DV	0.4860	4.2413	0.1146	YES	
SEFF→DV	0.0740	0.5331	0.1388	NO	
**FATHERS-DIRECT AND INDIRECT EFFECTS**					
EGRO→VIS	−0.007	0.0705	0.0993	NO	DIRECT
VIS→DV	0.6350	6.0041	0.1058	YES	
EGRO→DV	−0.2560	2.1170	0.1209	YES	
IGRO→VIS	−0.0180	0.1244	0.1447	NO	NONE
VIS→DV	0.6350	6.0041	0.1058	YES	
IGRO→DV	−0.1530	1.1804	0.1296	NO	
SX→VIS	0.2280	2.1204	0.1075	YES	INDIRECT
VIS→DV	0.6350	6.0041	0.1058	YES	
SX→DV	0.0430	0.4168	0.1032	NO	
SEFF→VIS	0.4610	1.9556	0.2357	YES^*^	INDIRECT
VIS→DV	0.6350	6.0041	0.1058	YES	
SEFF→DV	0.1330	1.0690	0.1244	NO	

* *Borderline significance*.

The results in Table 10 indicate a significant indirect effect between Self-efficacy/Perceived Daughter Efficacy and Daughter Succession in both the Daughters' and Fathers' models. Daughter Succession Vision connects these two variables, suggesting that Daughter Succession Vision is necessary before Self-efficacy/Perceived Daughter Efficacy influences Daughter Succession. Additionally, Daughter Succession Vision has a direct effect on the relationship between Sexism and Daughter Succession in the Fathers' model. This contrasts with the role of Daughter Succession Vision as a mediator in the Daughters' model. However, both the Daughters' and Fathers' models show Daughter Succession Vision as a strong influence between Sexism and Daughter Succession, suggesting that Sexism contributes to perceptions of daughters as successors in the family business. Instrumental Gender Role Orientation (IGRO) is not significantly related to Daughter Succession Vision or Daughter Succession. This conclusion agrees with the initial hypothesis test. Finally, a clear distinction is seen between Daughters' and Fathers' view of Expressive Gender Role Orientation. EGRO does not influence Daughter Succession in the Daughters' model but significantly affects Daughter Succession in the Fathers' model, indicating that fathers who perceive expressive qualities in their daughters do not consider their daughters as candidates for succession. This relationship had not been hypothesized.

### Comparative analysis

A Paired Sample *T*-Test was also conducted to understand differences within father/daughter pairs. Table 11 illustrates results of tests of hypothesized comparisons between daughters' and fathers' responses. We proposed that daughters believe they have more self-efficacy than fathers perceive (Hypothesis 1c) and daughters believe there is more sexism in society than fathers believe (Hypothesis 2c). Additionally, we posited that daughters believe they have stronger instrumental gender role orientations than expressive gender role orientations and fathers believe the reverse about daughters (Hypothesis 3f). Finally, we hypothesized that daughters' succession visions are stronger than fathers perceive (Hypothesis 4).

**Table 11:** **Paired sample *T*-Test results**.

**Hypothesis**	**Construct**	**Mean**	***t*-value**	***p*-value**	**Hypothesis supported**
H1c	Perceived Daughter Efficacy/Self-Efficacy	−0.083	−0.689	0.494	No
H2c	Sexism	0.211	2.483	0.020^*^	Yes
H3c	Expressive Gender Role Orientation	−0.025	−0.226	0.822	No
H3f	Instrumental Gender Role Orientation	−0.010	−0.085	0.933	No
H4	Vision	−0.267	−2.250	0.030^*^	Yes

* *Significant at 0.05 level*.

Table 11 shows that father and daughter pairs significantly differ in their average perceptions of Sexism in society. Based on the difference in means (Fathers minus Daughters), daughters see more sexism in society than their fathers, supporting hypothesis 2c. Father and daughter pairs also significantly differ in their perceptions of daughters' visions for the future of the business. The difference in means indicate that more daughters have greater succession visions than fathers perceive, supporting Hypothesis 4.

## Discussion

The purpose of this study was to understand how successors are chosen in family businesses and why so few daughters become successors. Specifically, we tested a set of propositions to determine the impact of self-efficacy/perceived daughter efficacy, gender beliefs, as indicated by beliefs about sexism in society and beliefs about gender roles, and shared vision on succession outcomes. The sample consisted of pairs of fathers and daughters because fathers are typically gatekeepers to leadership positions in family businesses. Three notable findings emerged from this study. First, the findings highlight the pivotal role of daughters' visions for the possibilities of the company. Second, the findings identify two differences between fathers' and daughters' beliefs suggesting misconceptions about daughters. Third, the study's results confirm the restraining role of gender biases in family businesses. Below we discuss each of these.

### The pivotal role of daughters' visions of possibilities for the company

Daughter Succession Vision served as a mechanism in our model to combine fathers' and daughters' perceptions of daughters' motivations and readiness to be successors. Informed by self-efficacy and gender beliefs, the vision construct allowed an evaluation of daughters' perceptions of the family business's purpose and calling as well as their fit with an image of a family business leader. Our construct, “Daughter Succession Vision,” encompassed shared beliefs and attitudes about the future of the family business and the likelihood that daughters would become successors. Results indicated that when daughters developed a vision for the future of the company and fathers recognized and shared their vision, daughters were more likely to become successors.

### Differences between fathers' and daughters' beliefs about vision and sexism

Data indicated a gap between fathers' and daughters' perceptions of daughters' views of the future of the company. The difference suggests that more daughters have visions for the future of the company than dads may realize. Extant literature describes daughters as “invisible” (Jimenez, 2009). Their role and contributions to the family business are subtle and unrecognized. Our data suggest that daughters' visions for the family business may be invisible to fathers and, as the antecedent to daughter succession, fathers' blindness to daughters' visions may be a restricting influence to daughter succession.

The findings of this study also indicate differences between fathers' and daughters' beliefs about discrimination toward women in society, with fathers believing there is less sexism than daughters perceive. This suggests subtle sexism (Benokraitis, 1997) as fathers may not be mindful of the restricting influences of mixed messages that encourage daughters toward achievement but do not offer necessary support.

### The role of gender biases

This research study indicates that daughters' visions of possibilities for the future of the family business may counteract restricting influences of gender biases. However, two factors confirm the presence of binding gender influences. The variable, Sexism, is an attenuating variable in our model, exerting a negative influence on the mediator and dependent variable. Findings that sexism is positively related to Daughter Succession Vision and Succession indicate that sexism reduces daughters' visions and succession. Daughters who perceive strong boundaries for females are less likely to prepare for succession or develop a vision for the family business.

Perhaps the most important evidence of gender bias was the finding of the negative relationship between fathers' beliefs about expressive behaviors and daughter succession. This relationship reveals the influence of sex stereotypes on perceptions of appropriate career choices for daughters. Fathers who perceived that daughters were expressive- or nurturing, caring, and cooperative- ruled out daughters' possibilities for succession. Gender biases therefore continue to affect the selection and self-selection of family business successors by impacting both fathers' and daughters' cognitions about women's roles in society.

## Conclusion

**Figure 2:**
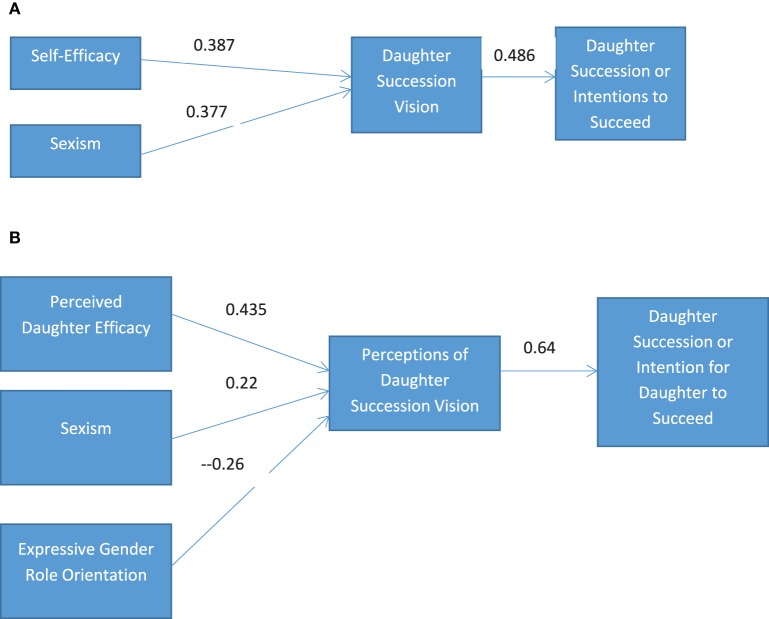
**Final Models. (A)** Daughters' final model. **(B)** Fathers' final model.

Results of the present study suggest the transformational qualities of shared vision. Shared vision not only transformed daughters into successors but may have helped daughters surmount gender barriers. Mediation, direct and indirect effects of shared vision revealed a process through which individuals self-select and are selected by others. Figure 2A indicates that self-selection is driven by self-efficacy, or daughters' beliefs that they have the abilities to lead the business. In turn, self-efficacy encourages daughters to develop an ideal vision for the business. However, sexism is an attenuating variable that mitigates daughters' visions for the future of the business. Likewise, Figure 2B shows that perceptions of daughters' efficacy to lead the family business as well as perceptions that daughters' have an agreeable vision for the future of the company, encourages fathers to select her for leadership. Conversely, gender biases serve as barriers to daughters' self-selection and fathers' selection of daughters as family business leaders. Daughters who perceive a discriminatory or sexist environment are not likely to develop a vision for the business. Importantly, daughters did not believe that gender role orientations influenced their family business leadership potential. Their beliefs about accepted roles for females and males were unrelated to the development of a vision for the business. Daughters' perceptions of a gender based discriminatory environment, however were factors that discouraged daughters from developing a vision. Beliefs about a sexist society are thus barriers to daughter leadership. Similarly, fathers' selections or dismissals of daughters for succession were influenced by gender biases. Fathers' perceptions of daughters' expressive characteristics disqualified daughters as successors. Furthermore, fathers blind to discriminatory environments did not perceive that daughters had a succession vision, the antecedent to succession. Data show that fathers' perceptions about who should lead the next generation of family businesses are impacted by undetectable but powerful gender influences. Thus, the key process for daughters to self-select and be selected as successors is to develop domain specific self-efficacy that is recognizable to others and to leverage that efficacy to form visions that might be shared by the current leader. Additionally, fathers' and daughters' awareness of the mitigating influences of gender biases can help them guard against these negative factors. In sum, understanding influences that lead to daughter inclusion or exclusion can help family business owners encourage and prepare their daughters for leadership in the next generation of their family business.

### Contributions to literature

Our study contributes to the theoretical literature by providing insights about the influence of Social Cognitive Theory on ICT. Our study indicated a strong relationship between self-efficacy and vision, a necessary component for change. Our study also shows linkages between Gender Theory and ICT, suggesting conditions for the activation of hopes and dreams for the future of the family business. Perceptions of gender inequality however, may suppress agency that leads to change. Finally, our study advances family business literature as it illuminates a process that is used to select next generation leaders. This process includes social cognitive variables integrated with desired goals or outcomes.

### Limitations

The use of a convenience sample where the geographical distribution of respondents is mostly from one area of the United States may limit the generalizability of these findings. A broader sample including the west and east coasts and larger cities may provide different results. Additionally, a larger sample of successor daughters may provide more insights into differences between successor and non-successor daughters.

Temporal effects potentially produce bias in fathers' responses. If daughters are in administrative positions fathers may be less likely to rate her as high on the perceived daughter efficacy scale than if the daughter were in an executive position. However, we argue that this potential bias is not relevant to family businesses. Fathers' assessments of daughters' abilities may cause daughters' current positions, not bias fathers' assessments after daughters' have assumed their positions. As chief leaders of the family business, fathers evaluate daughters' efficacy before appointing them to a position in the business. Therefore, daughters' positions or position titles do not likely impact fathers' assessments for this survey. For example, daughters in administrative positions may be there because fathers do not believe they can be effective executives or fathers may have placed them there to train for executive positions.

### Future research

The roles of self-efficacy and gender biases on the formation of a vision for the company suggest environmental layers or proximal and distal variables (Lent et al., 2000) that influence the development of a vision. These variables may provide more insight about the process of creating a vision and the role of personal perceptions and extra-person influences. Examining these dynamics in a family business might offer unique information due to the same actors in both the family and business systems.

Future research might also examine shared vision in the selection process of sons or other family members as successors in family businesses. A comparison between the selection processes of daughters and sons may yield more understanding of how shared vision may lead to family business continuity.

### Implications for practice

The power of shared vision to help daughters transcend gender barriers provides evidence for increasing communication between fathers and daughters. This study suggests that achieving shared vision requires fathers' awareness and understanding of daughters' career interests and the attenuating influences of gender biases. Communication is fundamental to this awareness and understanding. Fathers might also help daughters develop a vision for the family business by presenting opportunities for daughters to be involved in meaningful activities in the business. This exposure may boost daughters' domain specific self-efficacy, or self-efficacy relating to leading the family business. Mentoring actions would also be helpful to daughter succession. These may include psycho-social support (Kram, 1983) or introducing her to key players in the business such as managers, bankers, lawyers, accountants, suppliers and customers. In turn, daughters may enhance domain specific self-efficacy by taking deliberate steps to prepare to lead the family business. These activities may include taking business/management courses, gaining experience in business and in the specific industry, and developing a strategic perspective through exposure to business-wide decision making. They may seek nomination to serve on the boards of small companies or nonprofit agencies or join industry-specific associations and gain connections. Daughters may then leverage their business knowledge to develop a vision for the future of the company that they share with their father and others. Finally, daughters who perceive that their fathers have very strong gender biases might find support among professionals the father or other family members trust (Barnes, 1988).

### Conflict of interest statement

The authors declare that the research was conducted in the absence of any commercial or financial relationships that could be construed as a potential conflict of interest.
