# Supplementary material for: Shared vision between fathers and daughters in family businesses: the determining factor that transforms daughters into successors
Source: Front Psychol. 2015 May 29;6:625. doi: 10.3389/fpsyg.2015.00625 (PMC4448000; doi:10.3389/fpsyg.2015.00625)
Supplement: Supplementary file 1 [file DataSheet1.DOCX]

**APPENDIX A:
Measurement of Construct**

| Construct | Measurement Item | Source |
| --- | --- | --- |
| Gender Role Orientation | *Definition: Beliefs about the proper*  *Roles for men and women at work and at home.*  *Daughter Survey: The items listed below inquire about what kind of person you think you are. Please indicate how well each item describes you. Please choose one answer per item only.*  *Father Survey: The items below inquire about what kind of person your daughter is. Please indicate how well each item describes her. Please choose one answer per item only.*   1. Independent 2. Emotional 3. Active 4. Devoted to Others 5. Gentle 6. Helpful to others 7. Competitive 8. Kind 9. Aware of feelings of others 10. Decisive 11. Never gives up 12. Self-confident 13. Understanding 14. Warm 15. Stands up to pressure 16. Willing to take risks 17. Affectionate 18. Cheerful 19. Forceful 20. Has leadership abilities 21. Compassionate 22. Indifferent to others’ approval 23. Feelings not easily hurt 24. Superior | The Personal Attributes  Questionnaire (PAQ) (Spence & Helmreich, 1978,1979) |
| Successor Efficacy | *Definition: The combined beliefs of the successor and predecessor regarding the successor’s ability to execute the courses of action required to lead a profitable family businesses.*  *Daughter survey: An executive is a high level manager who is responsible and accountable for managing people, projects, and administrative functions. Suppose you are an executive in your family’s business. Please read the following statements and indicate how well each one reflects your beliefs about yourself.*  *Father survey: An executive is a high level manager who is responsible and accountable for managing people, projects, and administrative functions. Suppose your daughter is an executive in your family’s business. Please read the following statements and indicate how well each one reflects your beliefs about your daughter:*   1. My daughter will be able to achieve most of the goals that she sets for herself as an executive in our family business. 2. When facing difficult tasks within our family business, I am certain that my daughter will accomplish them. 3. In general, I think that my daughter can obtain outcomes that are important to her and the family business. 4. I am confident that my daughter can perform effectively on many leadership tasks in our family business. 5. Compared to other people, my daughter can do most executive tasks very well in our family’s business. 6. Even when things are tough, my daughter can perform quite well as an executive in our family’s business. | The General Self-Efficacy Scale (Chen, Gully, & Eden, 2001) |
| Daughter Succession  Vision | *Definition: A view of a desired leadership position to achieve a desired future of the family business.*  *Daughter survey: To what extent do you believe the following statements?*  *Father survey: To what extent do you believe the following statements regarding your daughter:*   1. I/My daughter has asserted a vision for the future of the family business. 2. I/My daughter often thinks about possibilities for the future of the family business. 3. I/My daughter believes the future of our family’s business will be better than the past. 4. I/My daughter feels inspired by the current visions and missions of our family’s business. 5. I/My daughter is encouraged by others to build on the strengths of our family’s business. 6. I/My daughter focuses on the vision or mission of the family business. 7. I/My daughter’s purpose is clear regarding the vision or mission of our family’s business.* 8. I/My daughter emphasizes the current strengths of our family’s business.*   *omitted from survey | PNEA Scale (Boyatzis & Oliver, 2008) |
| Sexism | Definition: Discriminatory practices against women including those that may be overt or subtle.  Daughter survey: To what extent do you agree or disagree with the following statements about American society?  Father survey: Same as daughter survey   1. Society has reached the point where women and men have equal opportunities for achievement. 2. Over the past few years, the government and news media have been showing more concern about the treatment of women than is warranted by women’s actual experiences. 3. On average, people in our society treat husbands and wives equally. 4. It is rare to see women treated in a sexist manner on television. 5. Women often miss out on good jobs due to sexual discrimination. 6. It is easy to understand why women’s groups are still concerned about societal limitations of women’s opportunities. 7. Discrimination against women is no longer a problem in the United States. 8. It is easy to understand the anger of women’s groups in America. | The Modern Sexism Scale (Swim & Cohen, 1997) |
| Intention | *Definition: Similar to Vision. Intention measures preparedness to act on a vision.*  *Daughter survey: Again, thinking about your family’s business, please indicate your level of agreement or disagreement with the following statements:*  *Father survey: Same as daughter survey*   1. I believe it is worthwhile for me/my daughter to be (become) an executive in our family’s business. 2. Based on her experience, I/my daughter is very likely to be an executive in our family’s business. 3. I/My daughter does not plan to be an executive in our family’s business now or in the future. 4. I will recommend that someone besides me/my daughter becomes an executive in my family’s business. | Online Learning Systems Scale (Lin, 2007) |

**Appendix B**

**Summary of Hypotheses Testing**

| **Hypothesis** | **Finding** |
| --- | --- |
| *Hypothesis 1a. Daughters’ self-efficacy is positively associated with Daughter Succession Vision in family businesses.* | Supported |
| *Hypothesis 1b. Fathers’ perceptions of daughters’ efficacy is positively associated with Daughter Succession Vision in family businesses.* | Supported |
| *Hypothesis 1c Fathers and daughters beliefs about Successor Efficacy are significantly different, with daughters believing they have higher levels of Successor Efficacy.* | Rejected |
| *Hypothesis 2a. Duaghters’ beliefs that sexism is strong in American society has a strong impact on whether or not daughters form a Daughter Succession Vision* | Supported |
| *Hypothesis 2b. When fathers’ subtle or covert sexist beliefs and attitudes increase, the likelihood they perceive daughters have a succession vision decreases.* | Supported |
| *Hypothesis 2c. Daughters believe there is more sexism in society than do fathers.* | Supported |
| *Hypothesis3a. There is a negative association between Expressive Gender Role Orientation and Daughter Succession Vision as perceived by daughters.* | Rejected |
| *Hypothesis3b. There is a negative association between Expressive Gender Role Orientation and Daughter Succession Vision as perceived by fathers.* | Rejected |
| *Hypothesis3c. . Fathers’ and daughters’ perceptions of daughters’ Expressive Gender Role Orientation are significantly different, with daughters believing they have lower levels of expressiveness.* | Rejected |
| *Hypothesis 3d. . There is a positive association between Instrumental Gender Orientation and Daughter Succession Vision as perceived by daughters.*  *.* | Rejected |
| *Hypothesis 3e. There is positive association between Instrumental Gender Role Orientation and Daughter Succession Vision as perceived by fathers.*  *.* | Rejected |
| *Hypothesis 3f. Fathers’ and daughters’ beliefs about daughters’ Instrumental Gender Orientation are significantly different, with daughters believing they have higher levels of Instrumental Gender Role Orientation.* | Rejected |
| *Hypothesis 4. Daughters’ “Daughter Succession Visions” are stronger than fathers perceive.* | Supported |
| *Hypothesis 4a. Daughter Succession Vision mediates the effects of daughters’ assessments of*  *1. Self-Efficacy,*  *2. Sexism,*  *3. Expressive Gender Role Orientation, and*  *4. Instrumental Gender Role Orientation on Daughter Succession.* | Partial Mediation  Supported  Rejected  Rejected |
| *Hypothesis 4b. Perceptions of Daughters’ Succession Vision mediates the effects of fathers’ assessments of : 1. Perceived Daughter Efficacy,*  *2. Sexism,*  *3. Expressive Gender Role Orientation, and*  *4. Instrumental Gender Role Orientation on Daughter Succession* | Indirect Effect  Indirect Effect  Direct  Rejected |
| *Hypothesis 4c The stronger Daughter’s Succession Visions, the more likely they will associate with Daughter Succession.* | Supported |
| *Hypothesis 4d. Fathers who perceive daughters have strong Daughter Succession Visions will positively associate daughters with Daughter Succession.* | Supported |
